# Association of *FCRL3* Genetic Polymorphisms With Endometriosis-Related Infertility Risk: An Independent Study in Han Chinese

**DOI:** 10.1097/MD.0000000000001168

**Published:** 2015-09-04

**Authors:** Haiyan Zhang, Zhen Zhang, Guang Li, Surong Wang, Shiqian Zhang, Beibei Xie

**Affiliations:** From the Department of Gynecology, Affiliated Qilu Hospital of Shandong University, Jinan, China (HZ) and Gynecology Ward-1 (HZ, ZZ, BX); Department of Gastrointestinal Surgery (GL), and Gynecology Ward-3 (SW), Linyi City People's Hospital, Shandong Province, China.

## Abstract

The Fc receptor-like 3 (*FCRL3*) gene was reported to be linked to a variety of autoimmune diseases, including endometriosis-related infertility. However, this linkage has not been studied in Chinese population and there has been no meta-analysis on the interrelationship of *FCRL3* gene and endometriosis-related infertility. The aim of the study was to investigate the association between FCRL3 genetic polymorphisms and the risk of endometriosis-related infertility in Han Chinese, and a further meta-analysis was conducted to confirm our results.

Four single nucleotide polymorphisms (SNPs) (rs7528684 [FCRL3_3], rs11264799 [FCRL3_4], rs945635 [FCRL3_5], and rs3761959 [FCRL3_6]) on *FCRL3* gene were genotyped in a case–control cohort composed of 217 patients suffering from endometriosis-related infertility and 220 healthy controls using cleaved amplification polymorphism sequence-tagged sites (polymerase chain reaction–restriction fragment length polymorphism, PCR–RFLP). Odds ratio (OR) and its 95% confidence interval (CI) was used to evaluate the association quantitatively. Furthermore, a meta-analysis of previous studies including the present study was implemented through Stata 11.0 (Stata Corporation, College Station, TX).

We found an approximately 1.4-fold significantly increased frequency of the FCRL3_3 variant in women with endometriosis-related infertility over the controls (OR = 1.41 [95% CI = 1.08–1.84], *P* = 0.013). However, no significant difference was found between women with endometriosis-related infertility and controls for FCRL3_4, FCRL3_5, and FCRL3_6. Regardless of the symptoms and the revised classification of the American Society of Reproductive Medicine (rASRM) stage of endometriosis, there was a significant association between FCRL3_3 variant and an increased risk of endometriosis-related infertility. Meta-analysis of previous studies combined with the present study further confirmed the association between FCRL3_3 and the risk of endometriosis-related infertility.

In summary, the present study suggested that *FCRL3_3* variant was associated with an increased risk of endometriosis-related infertility, regardless of symptoms, and rASRM stage of the patients. Meta-analysis of previous studies combined with the present study further confirmed our results. Further large-scale studies in the future are warranted to explore the association between *FCRL3* genetic polymorphisms and endometriosis-related infertility, as well as other human diseases, in Asian and other ethnicities.

## INTRODUCTION

Endometriosis, a common and chronically estrogen-dependent gynecological disorder, primarily manifests itself in implantation, growth and development of endometrial tissues in the peritoneal cavity, which are supposed to develop themselves within uterine cavity.^1^ Approximately 10% to 15% of women at reproductive age suffer from endometriosis and their clinical symptoms include severe pelvic pain, heavy menstrual pain, irregular menstrual bleeding, pain during intercourse, or exercise.^2,3^ Furthermore, nearly 50% of endometriosis patients are persecuted by fertility problems, including infertility.^3^ To date, endometriosis could be explained by several etiopathogenesis, including implantation theory, defective immune system, genetic factors, etc. As endometriosis has a relatively high inheritability ratio of 51%, some researches regarding genetic risk factors, such as estrogen receptor-1 (*ESR1*), estrogen receptor-2 (*ESR2*), and luteinizing hormone beta-subunit (*LHB*) genes, for endometriosis-related infertility have been conducted.^4,5^ Even though several meaningful conclusions have been drawn in previous studies,^6–8^ the exact etiology of endometriosis remains unclear and standard treatment for endometriosis has been deficient so far.^9^ To sum up, considering the unfavorable outcomes of people who are affected by endometriosis and vague etiology of endometriosis-related infertility, it is of great significance to further explore genetic risk factors associated with endometriosis-related infertility.

Among the possible causes of endometriosis, it has been demonstrated that the deficiency in the immune system might act as an impediment to clear endometrial cells from pelvic cavity.^10^ There are also evidences that B lymphocytes produce specific antiendometrial autoantibodies and that Tregs, known as regulatory T-cells, are key regulators to guarantee a specific immune response against ectopic endometrial fractions.^11^ Therefore, the genetic factors that regulate the performance of B-cells and T-cells could be closely relevant to endometriosis and even endometriosis-related infertility.^12–14^ Recently, Fc receptor-like 3 (*FCRL3*), situated at 1q 21–23, was suggested as a novel risk factor of autoimmune disease, partly because the gene involves itself in tyrosine-based activation and inhibition motifs in its cytoplasmic domain.^12,15–17^ FCRL3, encoded by this gene, is a member of the immunoglobulin receptor family which has structural homology to the Fc receptor, and it is principally expressed in B lymphocytes of lymph nodes and germinal centers, which have been implied to play a significant role in the etiology of endometriosis via secreting autoantibodies.^18^ Furthermore, researches by Swainson et al^19^ and Nagata et al^20^ showed that the expression of *FCRL3* could also be detected in Treg cells, serving to restrict the proliferation and cytokine release of T-cells. It might be hypothesized that *FCRL3* is associated with combined effects of B-cells and Tregs within the autoimmune system. Emerging evidence has indicated significant associations of single nucleotide polymorphisms (SNPs) in *FCRL3* gene with several autoimmune diseases, including rheumatoid arthritis (RA), thyroid disease,^21^ systemic lupus erythematosus,^22^ and Graves’ disease.^23^ Moreover, it is proposed that the same susceptibility loci may lead to diverse autoimmune diseases.^24^ Hence, there might be an association between *FCRL3* gene and endometriosis, which has gradually attracted increasing attention.

Several studies have suggested that *FCRL3* genetic polymorphisms might play a significant role in the pathogenesis of endometriosis-related infertility in Brazilian and Polish population.^25–27^ However, the conclusion that *FCRL3* gene is associated with endometriosis might not be applied precisely to Chinese population due to diversity in phenotype heterogeneity, ethnic background, and the fact that endometriosis is a multifactorial disease.^25^ Therefore, the present study is aimed to investigate the association between *FCRL3* gene variations and the risk of endometriosis-related infertility in Han Chinese population firstly, and a meta-analysis was also performed to further confirm our results.

## METHODS

### Study Subjects

A case–control study was performed to confirm the relationship between common mutations of *FCRL3* gene and susceptibility of endometriosis-related infertility. Peripheral blood samples were obtained from 217 female endometriosis-associated infertility patients (mean age: 33.26 ± 5.71 years) and 220 fertile women (mean age: 32.79 ± 5.56 years) between January 2013 and December 2014 from Linyi City People's Hospital, China. Recruited subjects are selected among Han ethnicity and all of them have no genetic relationship with each other. They are native to Jiangsu Province. The cases and controls were well matched for age and body mass index (BMI) (all *P* > 0.05). In the research, patients with endometriosis were diagnosed by laparoscopy and histological examination, and women who did not get pregnancy after at least 12 natural cycles were regarded as infertile.^28^ Patients with acute or chronic disorders, especially autoimmune diseases, were excluded from the study. The stage of endometriosis was determined according to the revised classification of the American Society of Reproductive Medicine (rASRM).^29^ In this group, minimal/mild (stage I–II) endometriosis was found in 88 cases (40.6%) and moderate/severe (stage III–IV) endometriosis in 129 cases (59.4%). A pretested questionnaire about demographic data and clinical characteristics was compiled by experienced and trained interviewers for all participants. The peripheral blood samples and clinicopathologic information could not be acquired before they were approved by the ethical committee of Linyi City People's Hospital. All volunteers were of their own wills to sign informed consents prior to the commencement of the research.

### Genotyping

Genomic DNA was isolated from the peripheral blood of patients and controls by implementing a standard salting out procedure in strict accordance with a standard protocol.^30^ Determination of *FCRL3* genetic variants (rs7528684 C/T, rs11264799 A/G, rs945635 C/G, and rs3761959 A/G) was executed by cleaved amplification polymorphism sequence-tagged sites (polymerase chain reaction–restriction fragment length polymorphism, PCR–RFLP). Primers (GenScript, Piscataway, NJ 08854, USA) for multiplex-PCR (m-PCR) and multiplex extension were shown in Table 1, which were designed with usage of computer software MassARRAY™ Assay Design 2.0. The amplification reaction were conducted via a 5 μL reaction compound (QIAGEN, 40724 Hilden, Germany) containing 10 mM dNTP, 15 mM MgCl_2_, 4 μM reverse PCR primers, 4 μM forward PCR primers, and 5 U/μL Hotstar Taq. Then tubes were amplified in light of the next PCR conditions: a single cycle of initial denaturation for 15 min at 95°C; 45 cycles of 20 s denaturation at 95°C; 30 s annealing at 56°C, and 1 min extension at 72°C; and a final extension for 3 min at 72°C. Furthermore, in the primer extension reaction, shrimp alkaline phosphatase enzyme (Sequenom Inc., 3595 John Hopkins Court, San Diego, CA 92121, USA) was firstly employed to dephosphorylate excluded dNTPs from the amplification reaction, after which the MassEXTEND reaction was performed. The reaction mixture (Sequenom, Inc.) was involved with 0.18 μL MassEXTEND primers (50 μM each), 0.2 μL hME EXTEND Mix (50 μM each, containing buffer and d/ddNTPs), and 0.04 μL Thermo Sequence™ (32 U/μL). And the cycling conditions for obtaining allele-specific extended products were described as follows: 94°C for 2 min, followed by 100 cycles of 94°C for 5 s, 52°C for 5 s, and 72°C for 5 s. Finally, approximately 10 μL desalted PCR products were placed on SpectroCHIP (Sequenom, Inc.) and they were analyzed entirely automatically with MassARRAY system (Bruker-Sequenom, San Diego, CA), which was devised on the basis of matrix-assisted laser desorption/ionization time-of-flight mass spectrometry (MALDI-TOF-MS) technology.

**TABLE 1:**
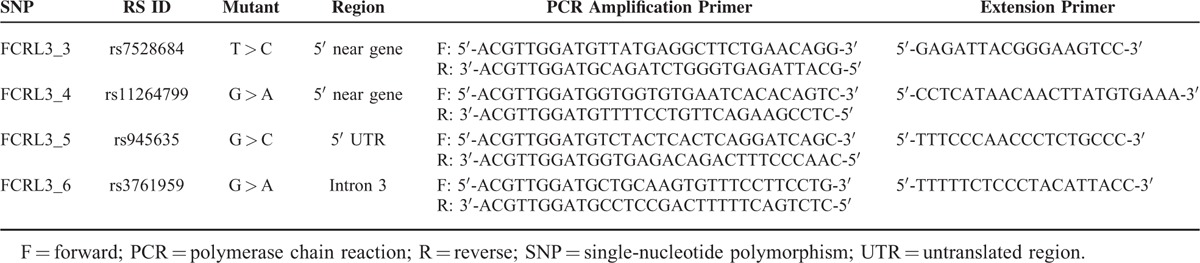
Primers of *FCRL3* Genetic Polymorphisms for PCR Amplification

### Statistical Analysis

Chi-squared (χ^2^) test was utilized to assess if statistical significance for differences in the frequency of alleles between patients and controls and deviation of the genotype frequency distribution from Hardy–Weinberg expectations existed. The Student *t* test and χ^2^ test were conducted to compare demographic characteristics between patients and controls, such as age of menarche, menstrual cycle length, menstrual period length, smoking status, family history of endometriosis, and sex hormone levels and symptoms. Odds ratios (ORs) adjusted by clinical parameters and their 95% confidence intervals (CIs) were used to evaluate the interconnection between gene variations and the risk of endometriosis-related infertility. For meta-analysis, the I^2^ and Cochran's Q test served to evaluate heterogeneity of between-studies. In the condition of I^2^ < 50% and *P* ≥ 0.05 for the Q test, the fixed-effects model was applied to integrate the data; otherwise, the random-effects model was employed instead. Statistic analyses were conducted using the Stata version 11.0 (Stata Corporation, College Station, TX), and a 2-tailed *P* value <0.05 was considered to be statistically significant.

## RESULTS

### Sample Characteristics

A total of 437 women were included in the study (217 endometriosis-related infertility patients and 220 fertile women in the control group). The median infertility duration of patients was 5.28 years. Clinical characteristics and hormonal parameters of the 2 groups were presented in Table 2. Most patients were in advanced stages (stage III and IV, 59.4%). No significant differences were found in some indexes between cases and controls, such as age, BMI, age of menarche, menstrual cycle length, menstrual period length, smoking status, and serum concentration of 6 sex hormones in advanced stages of the disease (all *P* > 0.05). However, higher frequency of infertile family history (*P* < 0.001) was observed for cases compared with controls. In addition, regarding to clinical symptoms, a significant number of cases reported chronic pelvic pain, dysmenorrhea, and dyspareunia. Though endometriosis symptoms were also reported by controls, significant differences were observed between the 2 groups in the prevalence of all symptoms (all *P* < 0.001). Therefore, they were adjusted in the multivariable logistic regression models for analyses of the association between *FCRL3* genetic polymorphisms and the risk of endometriosis-related infertility.

**TABLE 2:**
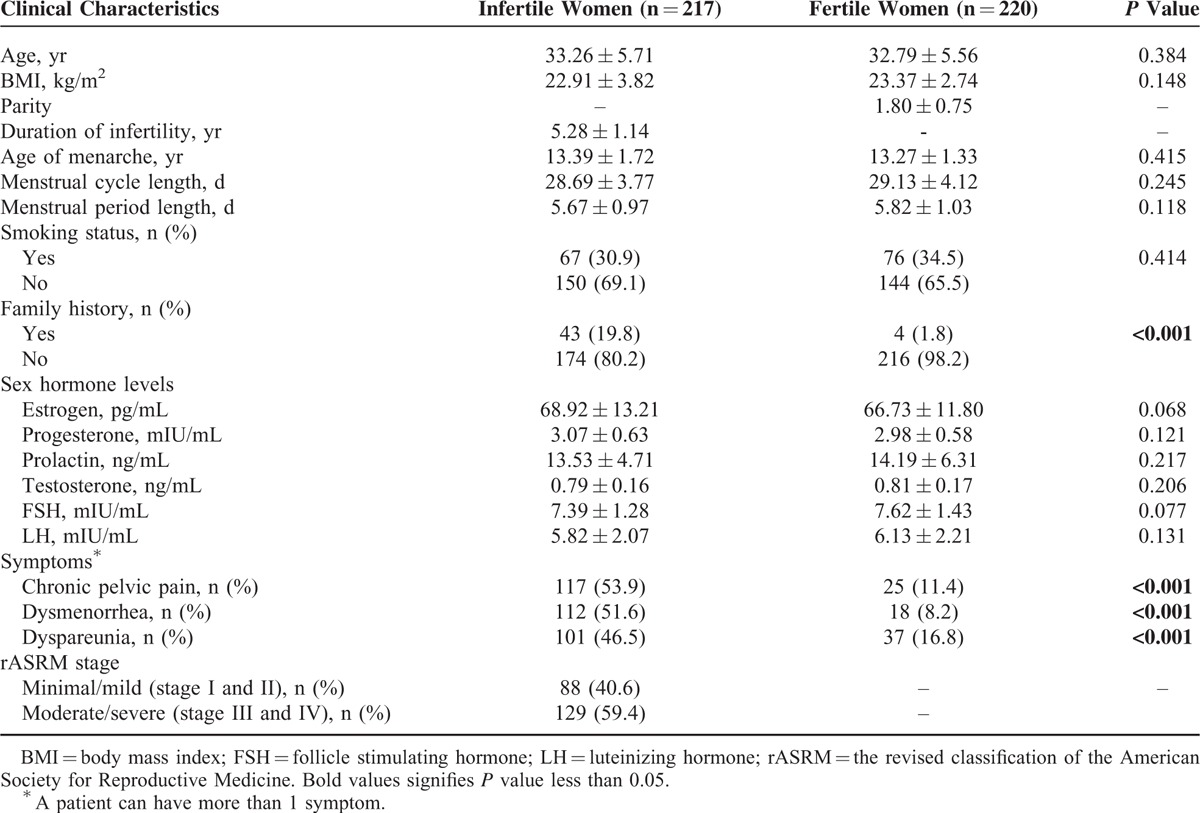
Comparison of Endometriosis-Related Infertility Patients and Controls by Selective Clinical Characteristics

### Association Between *FCRL3* Genetic Polymorphisms and Susceptibility of Endometriosis

**FIGURE 1:**
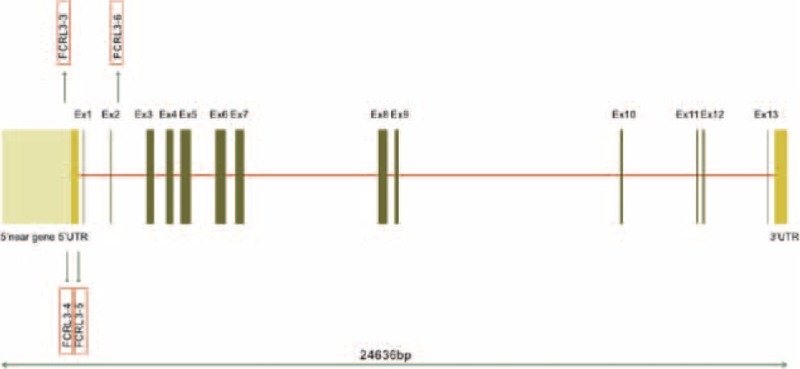
Genetic location of the 4 tag-single nucleotide polymorphisms in *FCRL3* gene.

As shown in Figure 1, FCRL3_3 and FCRL3_4 were located in the 5′ near gene region, whereas FCRL3_5 and FCRL3_6 were, respectively, situated in the 5′ untranslated region (UTR) and intron 3.^31^ The observed frequencies of each SNP met the assumptions of Hardy–Weinberg equilibrium (HWE) in both the case and control subjects (all *P* > 0.05). The results of the allele frequency analysis are presented in Table 3. We found an approximately 1.4-fold significantly increased frequency of the FCRL3_3 C allele in women with endometriosis-related infertility over the controls (allelic model: OR = 1.41 [95% CI = 1.08–1.84], *P* = 0.013). There was also a significantly increased frequency of the C/C and C/T genotypes in patients compared with controls (dominant model: OR = 1.69 [95% CI = 1.12–2.56], *P* = 0.012). With regard to the FCRL3_4 (rs11264799) polymorphism, no difference was found between women with endometriosis-related infertility and controls. Similar results were found for the FCRL3_5 (rs945635) and FCRL3_6 (rs3761959) polymorphisms.

**TABLE 3:**
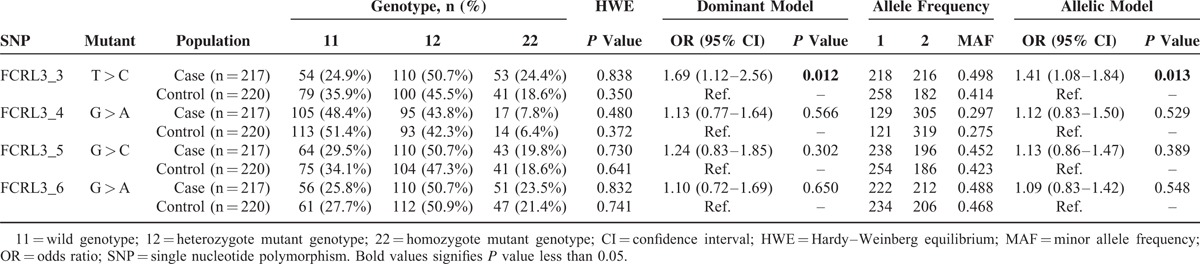
Allele and Genotype Distributions of *FCRL3* Genetic Polymorphisms in Endometriosis-Related Infertility Patients and Controls

To investigate whether there were other factors that might influence the overall results, further stratified analyses were conducted according to the clinical symptoms and rASRM stage (Table 4). Regardless of the symptoms of the disease, there was a significant association between FCRL3_3 variant and an increased risk of endometriosis-related infertility, which was also consistent with the overall analysis. When the case and control groups matched with the rASRM stage, the difference was more evident in the group of stage III–IV (C allele vs T allele: OR = 1.42 [95% CI = 1.04–1.93], *P* = 0.027; CT + CC vs TT: OR = 1.63 [95% CI = 1.01–2.64], *P* = 0.046; CC vs TT: OR = 1.93 [95% CI = 1.04–3.56], *P* = 0.035). For the subgroup of stage I–II, only a moderately significant association between FCRL3_3 variant and an increased risk of endometriosis-related infertility was found under dominant model (CT + CC vs TT: OR = 1.79 [95% CI = 1.01–3.14], *P* = 0.041).

**TABLE 4:**
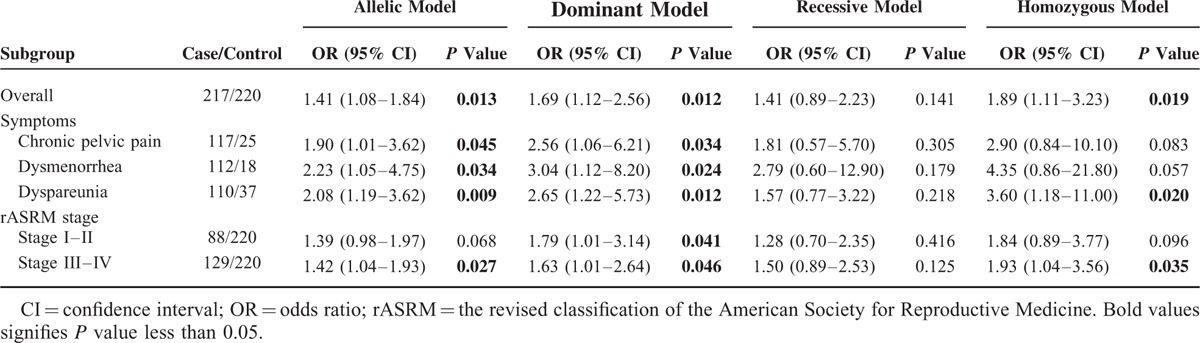
Stratified Analyses of FCRL3_3 (rs7528684) in Endometriosis-Related Infertility Patients and Controls

### Meta-Analysis of FCRL3_3 With the Risk of Endometriosis-Related Infertility

**FIGURE 2:**
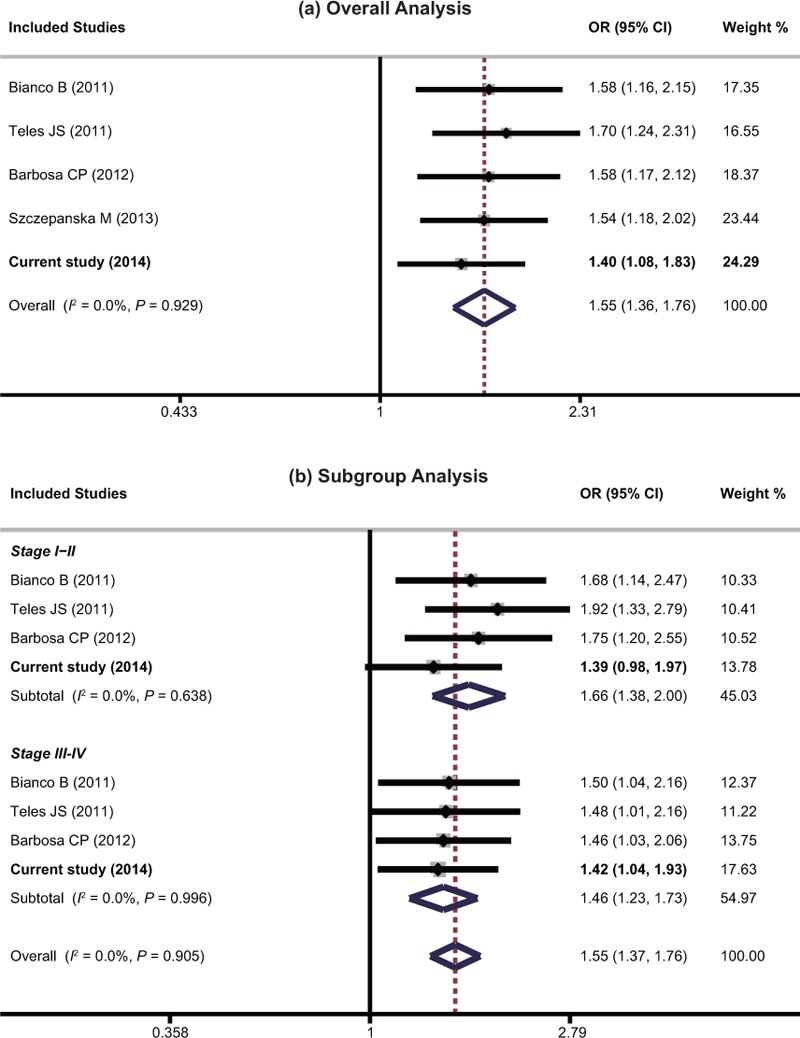
Meta-analysis with the allelic model (C vs T) and fixed effects for the association between FCRL3_3 and the risk of endometriosis-related infertility. The name of the first author and year of publication of each study are shown. The results of individual and summary odds ratio (OR) estimates, 95% confidence interval (CI), and weight (W) of each study show the association between FCRL3_3 and the risk of endometriosis-related infertility. Horizontal lines represent 95% CI and vertical-dotted lines represent the value of the summary OR.

Meta-analysis of 4 previous studies combined with the present study, including 883 cases and 881 controls, was implemented to confirm the association between FCRL3_3 and the risk of endometriosis-related infertility.^8,25,27,32^ Detailed information of included studies was enumerated in Table 5. As shown in Figure 2, the fixed-effects model was used due to low level of between-studies heterogeneity (I^2^ = 0%, *P* = 0.929). The overall analysis suggested that FCRL3_3 variant was associated with a significantly increased risk of endometriosis-related infertility under allelic model (OR = 1.55 [95% CI = 1.36–1.76], *P* < 0.001). Moreover, subgroup analyses were further performed based on the rASRM stage, which classified the patients into stage I–II (minimal/mild endometriosis) and stage III–IV (moderate/severe endometriosis) groups (Table 5). A total of 506 cases and 772 controls as well as 573 cases and 722 controls were, respectively, recruited into subgroup stage I–II and stage III–IV. Regardless of the rASRM stage, there was a significant association between FCRL3_3 variant and an increased risk of endometriosis-related infertility, which was consistent with the overall analysis.

**TABLE 5:**
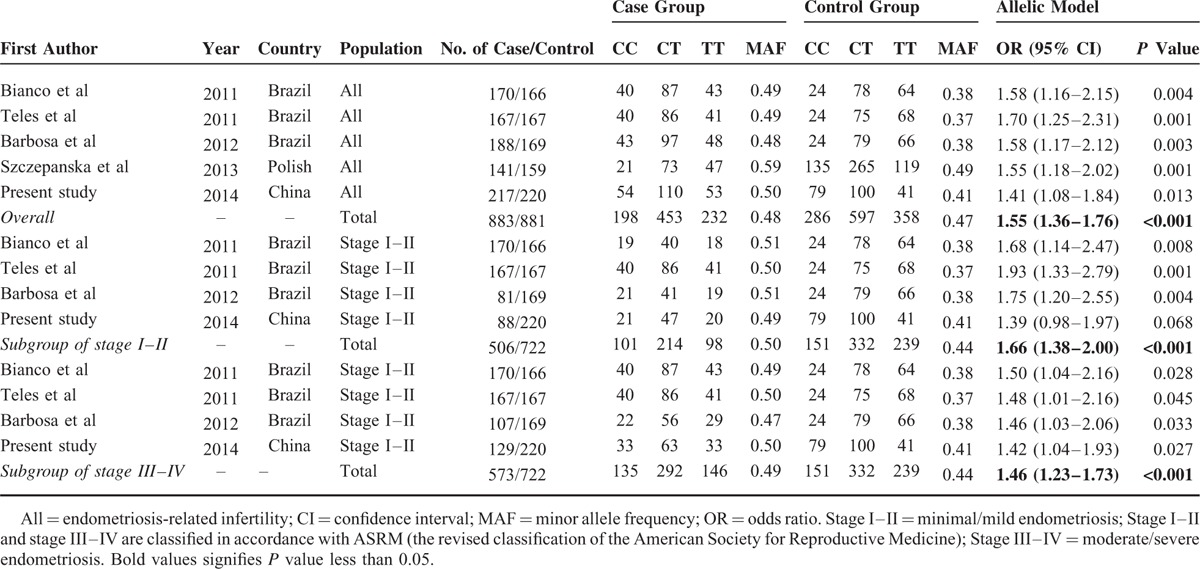
Meta-Analysis of FCRL3_3 (rs7528684) in Endometriosis-Related Infertility Patients and Controls

## DISCUSSION

Endometriosis-related infertility has been reported to co-occur with autoimmune diseases,^33^ while *FCRL3* genetic polymorphisms discourage regular expression of *FCRL3* in B cells, Treg cells, macrophages, etc., during the autoimmune process, indicating that *FCRL3* genetic polymorphism might be associated with the presence of endometriosis-related infertility. An association was observed between a short segment composed of 4 SNPs (FCRL3_3, FECRL3_4, FCRL3_5, and FCRL3_6) in *FCRL3* and RA.^21^ Simmonds et al^28^ also found that mutations of FCRL3_3 (OR = 1.17 [95% CI: 1.02–1.34]), FCRL3_5 (OR = 1.18 [95% CI: 1.04–1.35]), and FCRL3_6 (OR = 1.20 [95% CI: 1.05–1.36]) could heighten susceptibility of Graves’ disease by means of altering pathways of B cell signaling and activation. Furthermore, it has been identified by 4 whole-genome studies that overlapping cluster of susceptibility loci could bring about clinically distinct diseases, including RA,^34^ Graves’ disease,^35^ multiple sclerosis,^36^ and Type 1 diabetes.^37^ Thus, the 4 SNPs were selected for the study to investigate the association of *FCRL3* genetic polymorphism with risk of endometriosis-related infertility in Han Chinese population and a meta-analysis of previous studies together with the present study was implemented to further confirm our results, concluding that FCRL3_3 may serve as a susceptible factor for endometriosis-related infertility.

**FIGURE 3:**
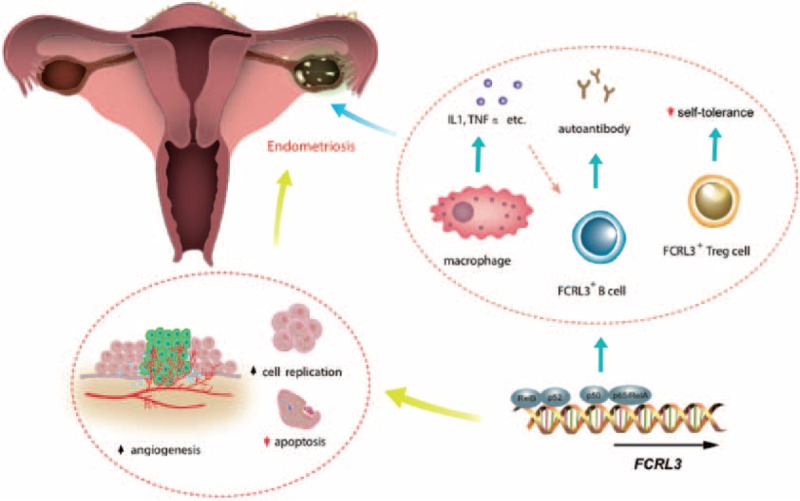
Potential mechanism of *FCRL3* gene in the pathogenesis of endometriosis-related infertility. Abnormal NF-κB binding, affected by FCRL3_3, could lead to increased cytokines (IL1, TNFα, etc.) secreted by macrophages and incremental FCRL3 encoded by *FCRL3* in B cells, which would altogether abnormally activate B cells and an autoimmune disease onset, and also contribute to increased angiogenesis, constitutive cell replication, and inhibition of apoptosis. Eccentric *FCRL3* expression in Treg cells, attributable to abnormal NF-κB binding, also served to disable regulatory activities of the cells. The combined effects of above indications ultimately made pelvic pain and endometriosis-related infertility possible. FCRL3 = Fc receptor-like 3.

In the present study, FCRL3_3 was the only genetic polymorphism to be remarkably associated with endometriosis-related infertility among the 4 SNPs. It might be due to the exceptional locus of FCRL3_3. In fact, FCRL3_3 was seated in the promoter gene of *FCRL3* and it was found to alter nuclear factor kappa-light-chain-enhancer of activated B cells (NF-κB) binding, which played a determinant role in the expression of *FCRL3*, both in vitro and in vivo,^38^ whereas no study has so far demonstrated how FCRL3_4, FCRL3_5, FCRL3_6 could influence *FCRL3* transcription. As shown in Figure 3, abnormal activation of NF-κB, which was a transcriptional factor and was made up of Rel B, p52, p50, and p65/RelA, might lead to expression of genes involved in inhibition of apoptosis, constitutive cell replication, increased angiogenesis, as well as incremental proinflammatory cytokines secreted by macrophages, all of which were indications of a defective immune system.^39^ Additionally, the cytokines, such as interleukin 1 (IL1), tumor necrosis factor α (TNFα), etc., also promoted deregulated activation of B cells.^40,41^ Furthermore, eccentric NF-κB binding would also contribute to increased *FCRL3* expression in B cells and thus signaling pathways were activated or inhibited, disturbing normal B cell functions.^18^ The defective immune system as mentioned above, including abnormal B cell activation, would consequently contribute to pelvic pain and subfertility.^42^ Besides, it was recently reported that *FCRL3* could also be expressed on natural killer cells and regulatory T cells (Treg) in addition to B cells,^20,43^ especially that *FCRL3* positive Tregs have failed to regulate their own activities and could no longer remain self-tolerant.^20^ Significant reduction of Tregs in eutopic endometrial tissues was observed in women without endometriosis during secretory phase compared with women with the disease, indicating that the increased *FCRL3* positive Tregs discourage novelly recruited immune cells from effectually identifying and aiming at endometrial antigens in the process of menstruation, enabling implantation of shed endometrial cells and possible infertility.^11^

Interestingly, the presence of endometriosis-related infertility is accompanied by clinical symptoms as chronic pelvic pain, dysmenorrheal, and dyspareunia, among which dyspareunia is the most significant when associated with FCRL3_3. The phenomena could possibly be explained by the long-periodic occurrence of chronic pelvic pain and dysmenorrhea (regularly once a month) in comparison with dyspareunia. In addition, patients with moderate/severe endometriosis (stage III–IV) were supposed to be more susceptible to infertility than stage I–II (minimal/mild endometriosis) counterparts. A possible explanation could be that patients with advanced stages of endometriosis revealed more serious clinical symptoms and larger possibility of infertility under the same frequency of FCRL3_3 variant.

To our best knowledge, this was the foremost case–control study in China about the association of *FCRL3* genetic polymorphisms with endometriosis-related infertility risk, in which 2 stratified analyses were also firstly conducted, respectively, in accordance with clinical symptoms (chronic pelvic pain, dysmenorrheal, and dyspareunia) and rASRM stage. Moreover, sample size of case subjects in the present study was relatively bigger than mean sample size of previous studies, partially leading to more convincing results that FCRL3_3 mutation may promote the occurrence of endometriosis-related infertility, especially for patients with moderate/severe endometriosis. Finally, the first meta-analysis of previous studies coupled with the present study was implemented to further confirm our results, revealing that our conclusion in Chinese population is consistent with antecedent outcomes within Brazilian and Polish population.

However, the excessive consistence in results between our study and previous studies might be attributed to limited sample size in each ethnicity that has been investigated, in which characteristics of the selective subjects may not suffice to be representative of those of the overall population within a certain ethnicity. Furthermore, it was untenable to equate Chinese population with other ethnicities on the association of *FCRL3* with endometriosis-related infertility owning to extra possible etiology of endometriosis-related infertility, such as discrepancy in environmental factors, individual genetic, lifestyle, reproductive risk factors, etc.^44,45^ Therefore, to further substantiate the effect of *FCRL3* mutation on susceptibility of endometriosis-related infertility in Asians and other ethnicities, large-scale and in-depth studies are in huge need.

In summary, the present study suggested that FCRL3_3 variant was associated with an increased risk of endometriosis-related infertility, regardless of symptoms and rASRM stage of the patients. However, this association was more evident in the group of stage III–IV patients and patients with dyspareunia symptom. Meta-analysis of previous studies combined with the present study further confirmed our results. Further large-scale studies in the future are warranted to explore the association between *FCRL3* genetic polymorphisms and endometriosis-related infertility, as well as other human diseases, in Asian and other ethnicities.
